# Diagnosis and Management of a Cardiac Amyloidosis Case Mimicking Hypertrophic Cardiomyopathy

**DOI:** 10.7759/cureus.3749

**Published:** 2018-12-18

**Authors:** Yasar Sattar, Tania Ruiz Maya, Fnu Zafrullah, Nirav B Patel, Sharaad Latchana

**Affiliations:** 1 Internal Medicine, Icahn School of Medicine at Mount Sinai, New York, USA; 2 Cardiology, Icahn School of Medicine at Mount Sinai, New York, USA; 3 Internal Medicine, Steward Carney Hospital, Tufts University School of Medicine, Boston, USA; 4 Internal Medicine, Lasante Health, Jersey City, USA; 5 Internal Medicine, American University of Integrative Sciences, Tucker, BRB

**Keywords:** cardiac amyloidosis, hypertrophic cardiomyopathy, left ventricular outflow tract obstruction, multiple myeloma, al amyloidosis

## Abstract

Cardiac amyloidosis is an acquired heart disease secondary to the deposition of β-pleated amyloid proteins in heart tissue. Amyloid light chain (AL) amyloidosis is usually secondary to multiple myeloma and can rapidly deteriorate cardiac function, with high mortality. Up to 50% of AL patients have cardiac involvement presenting as heart failure, conduction abnormalities, and cardiomyopathies. One of the rare presentations is the likely simulation of disease with hypertrophic cardiomyopathies like left ventricular outflow tract (LVOT) obstruction due to the systolic anterior motion of the mitral valve and irregular septal hypertrophy secondary to amyloid deposits. We present a case of cardiac amyloidosis secondary to multiple myeloma who presented with dynamic LVOT obstruction resembling hypertrophic obstructive cardiomyopathy and complicated by acute pulmonary edema. These complicated cases can be initially treated for pulmonary edema with an elevation of the head of the bed, furosemide, and nitroglycerin intravenously. For multiple myeloma, chemotherapy was continued. Beta-blockers, calcium channel blockers and angiotensin-converting enzyme inhibitors, and aldosterone receptor blocker were avoided due to poor tolerability. After symptomatic control, the patient can likely be scheduled for septal myotomy and the placement of a pacemaker or implantable cardiac defibrillator to prevent any arrhythmias causing sudden cardiac death in these subsets of patients.

## Introduction

Cardiac amyloidosis is an acquired heart disease secondary to the deposition of β-pleated amyloid proteins. The classification and prognosis of amyloidosis depend on the type of amyloid deposits, such as amyloid light chain (AL) amyloidosis, transthyretin amyloidosis, and amyloid-A amyloidosis. AL amyloidosis is usually secondary to multiple myeloma and can rapidly deteriorate cardiac function with high mortality [1]. Up to 50% of AL patients have cardiac involvement presenting as heart failure, conduction abnormalities, and cardiomyopathies. Early diagnosis and management are of prime importance in the prognosis. A diagnosis of AL cardiac amyloidosis requires a constellation of symptoms, physical examination, electrocardiographic (EKG) imaging, and laboratory findings [2]. We present a case of cardiac amyloidosis secondary to multiple myeloma presented with pulmonary edema with a dynamic left ventricular outflow tract (LVOT) obstruction resembling hypertrophic obstructive cardiomyopathy (HOCM).

## Case presentation

A 67-year-old man with a past medical history of multiple myeloma, hyperlipidemia, and hypertension presented to the emergency department with two days of chest pain, shortness of breath, and orthopnea progressing to a single episode of loss of consciousness on exertion. He reported having similar symptoms over the past two weeks, but it has progressed in severity and had accompanied an episode of syncope in the last two days. Syncope is not preceded by any prodromal symptoms like dizziness, sweating, or autonomic symptoms. The patient denied fever, chills, nausea, vomiting, and abdominal pain.

On physical examination, he appeared alert and in no acute distress. His heart rate was 84 beats per minute; blood pressure was 120/70 mmHg; respiration rate was 28 breaths per minute, and peripheral capillary oxygen saturation was 98%. Cardiac examination showed elevated jugular venous pressure, a systolic ejection murmur at the apex and left of the sternal border. The respiratory exam showed a bilateral inspiratory crackle. The other findings of his examination were unremarkable.

**Figure 1:**
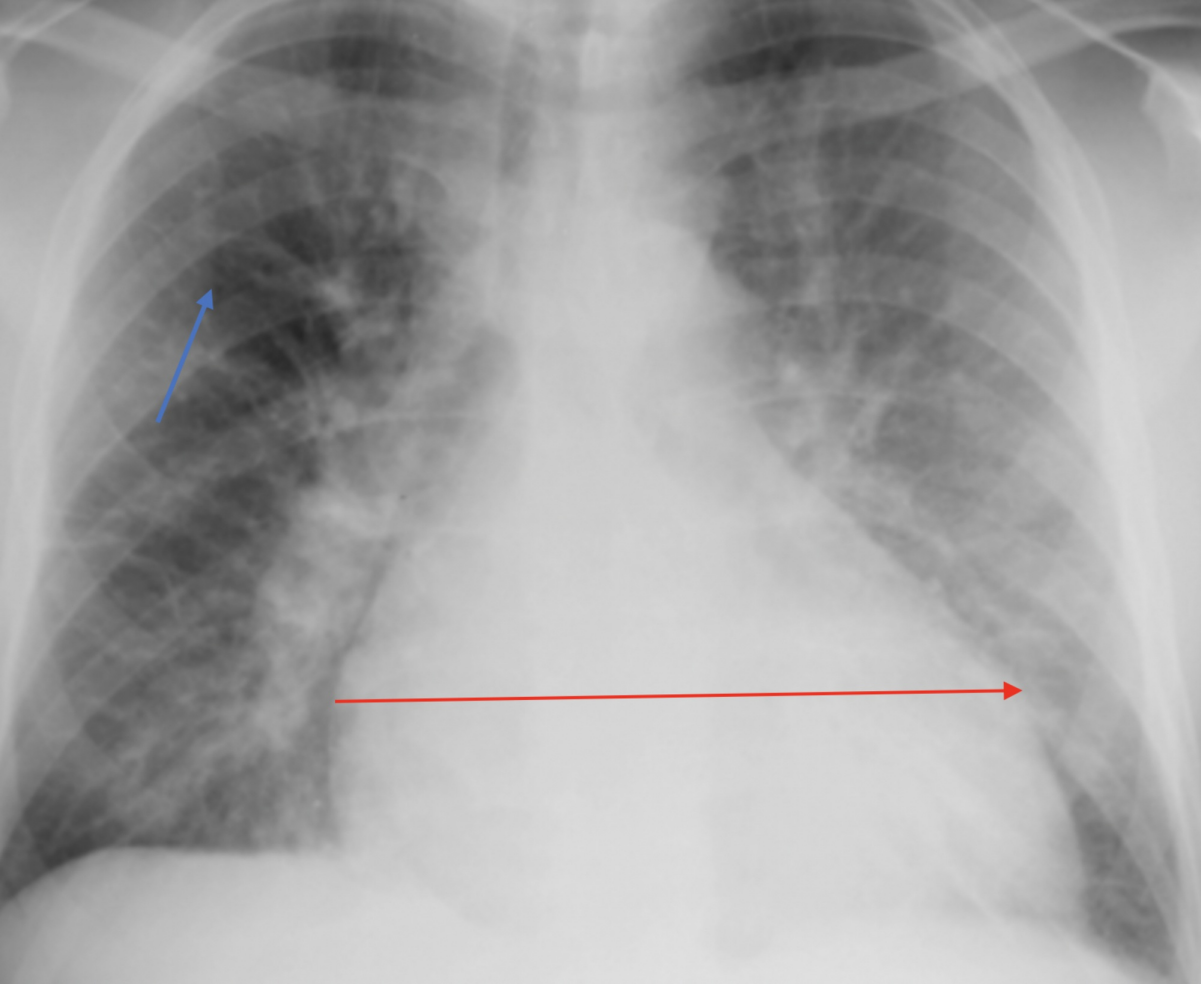
Acute pulmonary edema finding: blue arrow indicates Kerley B-lines and red arrow indicates significant cardiomegaly

Initial laboratory investigations showed normal white blood cell count, normal hemoglobin, and normal electrolytes. His pro-brain natriuretic peptide (BNP) was 1425 pg/mL (reference range, 1–100 pg/mL). Chest radiography showed pulmonary edema in the lower lung field of both lungs with some Kerley-B lines, as shown in Figure 1.

**Figure 2:**
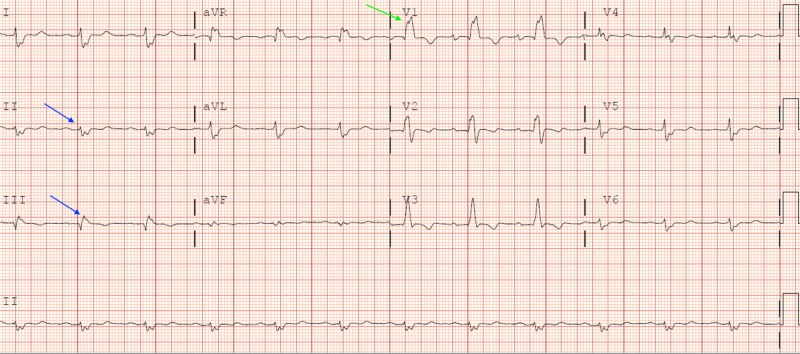
Blue arrow: sinus rhythm with low voltages in I, II, III limb leads; green arrow: rsR' in V1 (right bundle branch block)

EKG showed a right bundle branch block with low voltage in the limb leads and no ST-T ischemic change, as shown in Figure 2.

**Figure 3:**
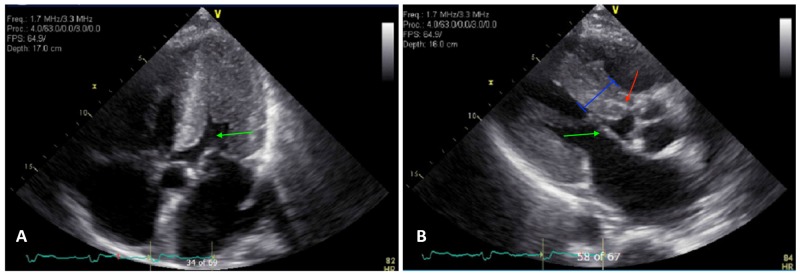
a) Green arrow: marked LVH with a narrow chamber; b) blue line: irregular septal hypertrophy with partial LVOT; green arrow: SAM causing LVOT; red arrow: speckled/granular appearance of the myocardium LVH, left ventricular hypertrophy; LVOT, left ventricular outflow tract; SAM, systolic anterior motion

Echocardiogram showed a markedly increased left ventricular (LV) wall thickness, hyperdynamic function with an ejection fraction of 75%, paradoxical motion of the interventricular septum with LVOT obstruction due to systolic anterior motion (SAM) of the mitral valve, and moderate mitral and tricuspid regurgitation, as shown in Figure 3. The results of genetic testing for HOCM mutations were negative.

A constellation of findings on laboratory, EKG, echocardiogram, and magnetic resonance imaging (MRI) in the setting of the history of multiple myeloma led us to a presumptive diagnosis of acute decompensated diastolic heart failure with LVOT obstruction secondary to cardiac amyloidosis. Initial symptomatic treatment started by an elevation of the head of the bed and one dose of furosemide 40 mg and nitroglycerin 200 mcg/minute intravenously. For multiple myeloma, we continued treatment with cyclophosphamide, thalidomide, and dexamethasone. Beta-blockers, calcium channel blockers, angiotensin-converting enzyme (ACE) inhibitors, and angiotensin II receptor blockers (ARB) were avoided due to concerns of symptomatic deterioration with these medications. After symptomatic control, the patient was scheduled for septal myotomy that helped in symptomatic improvement, and we placed an implantable cardiac defibrillator (ICD) to prevent any arrhythmias causing sudden cardiac death.

## Discussion

Cardiac amyloidosis refers to heart disease secondary to the deposition of amyloid fibrils in the heart. Amyloid deposition can present as restrictive cardiomyopathy, heart failure, and rhythm abnormalities due to conduction blocks and valvular involvement. Cardiac amyloidosis can present as both systolic and diastolic heart failure [2]. One of the rare presentations is a simulation of disease with hypertrophic cardiomyopathies like LVOT obstruction due to SAM of the mitral valve and irregular septal hypertrophy secondary to amyloid deposits [3]. Mostly cardiac amyloidosis resembling HOCM is found in the senile transthyretin type, but few cases of the AL type presenting as hypertrophic cardiomyopathy (HCM) are seen. It is very important to diagnose these patients early, and robust treatment is crucial for survival as the AL type cardiac amyloidosis has the worst prognosis. Diagnosis depends on a constellation of symptoms, physical examination, laboratory, imaging, and biopsy findings. Symptoms can range from fatigue, dyspnea on exertion, orthopnea, palpitations, and syncope [4]. Physical examination in CA can show mitral regurgitations, tricuspid regurgitation, or left ventricular outflow obstruction systolic murmur and jugular venous distention, as seen in our patient. Laboratory findings can show BNP > 400 pg/mL and high immunoglobulin levels. EKG findings include non-specific low voltage in limb leads seen in up to 50% of the patients, but patients can have a left bundle or right bundle branch block with pseudo-infarct Q-waves in lead V1-V3 [5]. Transthoracic echocardiography (TTE) is the usual, cheap, and non-invasive test of choice to diagnose cardiac amyloidosis. Early findings can include diastolic dysfunction with a high ejection fraction and increased left ventricular wall thickness. Other findings of TTE can include atrial enlargement, irregular septal hypertrophy, and speckled or granular myocardial appearance. Our patient had increased left ventricular thickness with diastolic dysfunction and an ejection fraction of 75%. Moreover, he also had SAM of the anterior mitral leaflet hitting the irregular septum, causing outflow obstruction, as shown in Figure 2 [6]. A disproportion of low voltage on EKG with left ventricular hypertrophy (LVH) on echocardiography is unique to cardiac amyloidosis and serves as an index of severity [7]. Cardiac MRI can show global transmural to sub-endocardial late gadolinium enhancement, rarely seen in other cardiomyopathies [8]. A biopsy can add to the clinical impression of amyloidosis and sources can include abdominal fat pad and endocardial biopsy showing amyloid fibrils positive for Congo-red stain. An endocardial biopsy is the gold standard method of diagnosis of cardiac amyloidosis, but it is invasive and requires great expertise [9]. After a presumptive diagnosis of cardiac amyloidosis with dynamic LVOT obstruction, it should be differentiated from HCM via genetic testing, although some cases of CA with positive genetic testing for HOCM have been documented.

The treatment of cases of cardiac amyloidosis with LVOT obstruction complicated by acute pulmonary edema involves the propped-up position, diuresis, nitrates, and spironolactone. Beta-blockers and calcium channel blockers are poorly tolerable in CA patients. ACE/ARBs can be used with caution due to symptomatic deterioration. Myomectomy can be considered in severe septal hypertrophy cases causing obstruction due to SAM. These patients have a high risk of developing life-threatening arrhythmias, so adding a pacemaker/ICD add a great prognostic benefit. Definitive treatment is cardiac transplantation for carefully selected candidates [10]. In all cases of cardiac amyloidosis secondary to amyloidosis, the chemotherapeutic management of multiple myeloma should be continued.

## Conclusions

Cardiac amyloidosis is a rare entity secondary to multiple myeloma. Cardiovascular symptoms in multiple myeloma can be likely considered as the deposition of amyloid fibrils in the heart. In rare circumstances, cardiac amyloidosis with LVOT obstruction can mimic HOCM. It should be differentiated based on genetic testing, and a disproportion of low voltage on EKG with LVH on echocardiography is unique to cardiac amyloidosis as a cause of septal hypertrophy causing LVOT obstruction. We presented this case report to highlight the different presentation of cardiac amyloidosis and to share our experience in managing this condition both symptomatically and therapeutically for multiple myeloma, as patients have very high mortality.

## References

[1] Falk RH (2005). Diagnosis and management of the cardiac amyloidoses. Circulation.

[2] Falk RH, Comenzo RL, Skinner M (1997). The systemic amyloidoses. N Engl J Med.

[3] Morner S, Hellman U, Suhr OB, Kazzam E, Waldenstrom A (2005). Amyloid heart disease mimicking hypertrophic cardiomyopathy. J Int Med.

[4] Quarta CC, Kruger JL, Falk RH (2012). Cardiac amyloidosis. Circulation.

[5] Lee MH, Lee SP, Kim YJ, Sohn DW (2013). Incidence, diagnosis and prognosis of cardiac amyloidosis. Korean Circulation J.

[6] Liu D, Niemann M, Hu K (2011). Echocardiographic evaluation of systolic and diastolic function in patients with cardiac amyloidosis. Am J Cardiol.

[7] Carroll JD, Gaasch WH, McAdam KP (1982). Amyloid cardiomyopathy: characterization by a distinctive voltage/mass relation. Am J Cardiol.

[8] Syed IS, Glockner JF, Feng D (2010). Role of cardiac magnetic resonance imaging in the detection of cardiac amyloidosis. JACC Cardiovasc Imaging.

[9] Pellikka PA, Holmes DR, Edwards WD, Nishimura RA, Tajik AJ, Kyle RA (1988). Endomyocardial biopsy in 30 patients with primary amyloidosis and suspected cardiac involvement. Arch Intern Med.

[10] Mookadam F, Haley JH, Olson LJ, Cikes M, Mookadam M (2006). Dynamic left ventricular outflow tract obstruction in senile cardiac amyloidosis. Eur J Echocardiogr.

